# Trauma Care in Low- and Middle-Income Countries

**DOI:** 10.1055/s-0041-1732351

**Published:** 2021-10-22

**Authors:** Dhurka Shanthakumar, Anna Payne, Trish Leitch, Maryam Alfa-Wali

**Affiliations:** 1 Department of Surgery, Whipps Cross Hospital, London, United Kingdom; 2 Department of Surgery, Royal London Hospital, London, United Kingdom; 3 Department of Surgery, St George's Hospital, London, United Kingdom

**Keywords:** trauma care, trauma, low- and middle-income countries

## Abstract

**Background** Trauma-related injury causes higher mortality than a combination of prevalent infectious diseases. Mortality secondary to trauma is higher in low- and middle-income countries (LMICs) than high-income countries. This review outlines common issues, and potential solutions for those issues, identified in trauma care in LMICs that contribute to poorer outcomes.

**Methods** A literature search was performed on PubMed and Google Scholar using the search terms “trauma,” “injuries,” and “developing countries.” Articles conducted in a trauma setting in low-income countries (according to the World Bank classification) that discussed problems with management of trauma or consolidated treatment and educational solutions regarding trauma care were included.

**Results** Forty-five studies were included. The problem areas broadly identified with trauma care in LMICs were infrastructure, education, and operational measures. We provided some solutions to these areas including algorithm-driven patient management and use of technology that can be adopted in LMICs.

**Conclusion** Sustainable methods for the provision of trauma care are essential in LMICs. Improvements in infrastructure and education and training would produce a more robust health care system and likely a reduction in mortality in trauma-related injuries.

Injuries contribute to more deaths globally each year than the combination of HIV/AIDS, malaria, and tuberculosis. 1 The global burden of injury is inversely proportional to income 2 3 ; therefore, it is not surprising that low- and middle-income countries (LMICs) suffer the largest volume of injuries. Unfortunately, LMICs lack robust trauma and emergency surgery care. 4 Trauma alone in LMICs is estimated to account for approximately 5 million annual global deaths. 5 6 7 Globally, ∼50 million individuals per year are left disabled after an injury. 5 In Africa, 38 per 1,000 disability-adjusted life years are reported compared with the global figure of 27 per 1,000. 8 9 This may be due to war, assaults, and vehicle accidents that primarily affect a young population. This problem is compounded by lack of rehabilitation facilities. Often, even when these services exist, cost, or inadequate and unskilled staff inhibits access. Following traumatic injury, early presentation to hospital is unusual and diagnostic imaging remains scarce, thereby worsening the prognosis in LMICs. The main purpose of this review is to identify current issues and solutions surrounding provision of trauma care in LMICs. There were three broadly identified challenging areas with trauma care in LMICs. These included infrastructure, education, and practical operational measures which are individually evaluated later.

## Methodology

PubMed and Google Scholar were used to identify relevant articles using the search MeSH terms “trauma,” “injuries,” and “developing countries.” Articles conducted in LMICs as defined by the World Bank classification, 10 discussing problems with the management of trauma patients, or articles that consolidated treatment and educational solutions about trauma care, published between 1990 and 2020, were included. Of 4,432 search results, 45 articles were included. Review of relevant articles enabled the identification of three main areas for improvement in trauma care in LMICs, based on the domains identified by the World Health Organization (WHO) on surgical care systems strengthening. 11

### Infrastructure

Trauma care systems save lives in high-income countries (HICs). 1 However, basic infrastructure is lacking in many LMICs. A key failure, with significant implications on public health in LMICs, is the lack of availability of basic amenities. Lack of water and electricity have a major impact on health care settings. 12 Problems range from the simple provision of clean drinking water and sanitation facilities, to the inability to clean hospitals and autoclave surgical instruments. 13 These problems inevitably have a role in the epidemiology of surgical site infections and hospital acquired infections in LMICs.

Funding for surgical care is limited or omitted completely in LMICs. 14 15 This may be lacking as surgical treatments are deemed expensive, and not as cost effective compared with dealing with communicable disease management. Despite these preconceived ideas, surgery does occur at low cost, with rates from $40 for a major surgical procedure in a low-volume hospital to $300 in a high-volume one. 14 However, the standard of care provided is variable and difficult to measure, as is the definition of what constitutes major surgery.

There is a need for structurally safe buildings as research from the WHO has shown. 11 Existing hospital buildings in LMICs are poorly maintained, with suboptimal management of clinical services. These should ideally be designed and constructed in a collaborative project with local and international architects and engineers. Materials could be sourced locally or used in combination with imported materials that would facilitate easy maintenance. Sustainability should be a focus, such as the use of solar panels for generating power to buildings, to help reduce long-term costs. Operating room facilities need to be updated. The provision of intensive care units would allow further management of the complex trauma patient and improve survival rates. Nevertheless, there needs to be a balance between resource availability and utilization to enable adequate and effective use of materials. This would facilitate comprehensive progress providing equipment and developing services that are beneficial in the LMIC setting, rather than just a drive to mirror HICs.

The poor condition of roads and vehicles, as well as nonadherence to road signs and speed limits, provides the formula for high numbers of road traffic injuries in LMICs. The causes are multifaceted and include poor road safety measures, inadequate roads, and human behavior. 16 The majority of traumatic injuries in LMICs are caused by road traffic collisions (RTCs). The death rate following RTCs in low-income countries (LICs) averages 27.5 deaths per 100,000 population compared with 8.3 per 100,000 in HICs. 17 Procurement of engineers with expertise in road planning, traffic flow control, and maintenance of these facilities are necessary developments to minimize the injuries caused by RTCs. There is also a need to establish safety standards for vehicles and mandatory annual car safety inspections together with accountability measures as in HICs where drivers are traceable. The mean costs of injury in LMICs has been reported to vary between $14 and $17,400, with the costs of preventative interventions being slightly less with the use of speed bumps 18 and other road safety measures. Other modes of transport, such as trains and ferries, could be used as alternative transport methods if proper training and safety measures were put in place, contributing to lowering the burden of RTC-related injuries. 19

Infrastructural challenges and problems with resource distribution also limit the adequate management of trauma in LMICs. 20 Organizational infrastructure such as fire services and safety measures may have an impact on the incidence and severity of burn injuries. Investment in the provision of adequate and affordable safe housing and living conditions to lessen overcrowding are essential considerations to be made on a national level in LMICs to avoid building accidents. Poor access to hospital services hinders injured patients from being treated promptly. Reasons for poor access include availability of transport vehicles, poor and unsafe traveling conditions, lack of nationalized systems to contact emergency services, and scarcity of emergency equipment. Prehospital care may be limited or not available, leading to more deaths outside the hospital in LMICs. 2 Innovative strategies for providing prehospital care have been utilized in LMICs such as the training of first responders among the lay public as well as taxi drivers. 21 22 23 Partnerships between governments and private sector companies form one potential strategy to address transportation problems. Change to the infrastructure in LMICs requires a radical approach that will include prevention strategies through injury surveillance, better prehospital and in-hospital care, as well as rehabilitation.

### Education

Guidelines and protocols have been associated with decreased morbidity and mortality in HICs. 24 Therefore, suitably developed guidelines need to be established in LMICs. Trauma educational programs in LMICs have been found to have a positive impact on practices and knowledge; however, translation into good patient outcomes is not described. 25 26 27 The provision of courses is not the sole important aspect of educational progress. Retraining and revalidation are crucial to the acquisition and development of skills along with the consolidation of knowledge. Skills training and continuing professional development should be emphasized within health care settings in LMICs.

Basic life support (BLS) training of health care professionals is lacking in many LMICs. The principle of prompt resuscitation needs to be embedded into the acute care of trauma patients, with training starting at undergraduate level. Training for BLS is relatively simple to set up and, as the equipment is reusable, annual recertification is easy to establish, as staff can be trained in-house. 28 The trauma management courses in HICs should be specifically tailored for use in LMICs. Low-cost alternatives to the Advanced Trauma Life Support (ATLS) courses have been developed, such as the Trauma Evaluation and Management course. 29 Equivalent courses to the Pre-Hospital Life Support course, tailored in an affordable manner, can be built into preexisting systems. In Nigeria and Ghana, trauma education courses have been developed from the ATLS course with the materials and infrastructure adapted to suit the population. 30 31 However, the continued practice and dispersion of these courses is yet proven, and evaluation in low-resource settings remains a continuous challenge.

The lack of adequate training, low salaries, and the brain drain of medical professionals to HICs contribute to the staff shortages in LMICs. 8 Several African countries have a high mortality rate associated with general anesthesia. 32 Some countries have only one or two trained anesthetists, with the remaining anesthetic cover being provided by nurses. Secondment of well-trained anesthetists from a global pool to sustain education, program development and training may be the way forward to counteract this issue. Countries such as Malawi and Mozambique combat medical personnel shortages by training nurses and medical assistants to perform surgical procedures 33 34 ; however, the data on outcomes are scarce. The concept of multidisciplinary team working is deep-rooted in HICs. This concept should be encouraged in LMICs, via colearning and simulation teaching, to establish the provision of coordinated care. This could be done at negligible cost to institutions in LMICs; thus, enhancing trauma team training, understanding of criteria for trauma calls, and improved triaging; as emergency departments are mostly nurse-led in LMICs.

Public health campaigns would be helpful in injury prevention. Campaigns and information sheets should be implemented in written and pictorial formats, and should be easy to understand without the need for literacy. Rehabilitated trauma patients should be advocates for education about safety, through the implementation of community collaborations with the understanding of legislation and law enforcement.

### Practical and Operational Measures

The management of the critically ill surgical patient requires prompt diagnosis of the injuries. The lack of diagnostic imaging modalities such as computed tomography is apparent in LMICs. 35 In some LMICs, radiographs are still processed using traditional techniques, with the drying of the films on washing lines in the sunshine. This is a slow and unsustainable process in the management of a trauma patient where time is of the essence. The type of trauma-related injury varies from neurological, musculoskeletal, visceral trauma and burns. Establishing simple trauma scoring systems that do not require complex data on anatomical injuries is beneficial, 36 such as the Kampala trauma score in Uganda. 37

Most LMICs have limited staff in terms of both health care professionals and supporting technical staff, such as laboratory staff and therapists. 38 39 It is however acknowledged that nonmedical personnel provide the surgical and trauma care in low-resource settings. 40 Algorithms for the initial assessment and reassessment of the critically ill surgical patient are essential. Setting realistic physical thresholds for trauma team activation in LMICs is vital for human resource planning. Work force planning is paramount in the future development of any country. A model of a fee-for-service, rather than salaried positions, could be tried by governments to try and minimize the brain drain of health care professionals from LMICs going to HICs.

There is a stark difference between LMICs and HICs with regard to the use of information technology and communications in the health care setting. The lack of universal availability of computers in public hospitals but the availability of mobile phones makes them amenable to use in hospitals. Trials using mobile technology as a tool to implement electronic-based health records have been positive. For example, the electronic Trauma Health Record (eTHR) made the use of tablet-based health records. This was both designed and tested in low-income settings. The eTHR included features to upload trauma admission data in terms of demographics, diagnosis, treatments, complications, and follow-up plans. 41 Such electronic registries enable accurate auditing and documentation of information regarding injury, allowing future improvement of services.

The benefit of mobile phone technology is still vastly underutilized and would be a useful source for obtaining trauma data. Remarkably, a high proportion of individuals in LMICs have mobile phones, thus can be used to enable prehospital communication and improve preparedness for hospital staff. Telemedicine has previously been applied to good use, for example, between the United States and Armenia immediately after disaster situations, which improved diagnostic accuracy and patient care in the immediate phase. 42

## Discussion

This article highlights some of the problems of trauma care provision in LMICs and offers possible solutions, which are by no means exhaustive. It is estimated that 2 million deaths per year could be prevented through the provision of adequate trauma care in LMICs. 43 The global socioeconomic impact of surgical conditions in terms of the number of days lost to working, percentage of unemployment, and the effect on families is likely to be underestimated. Governments of LMICs have an integral role to play to allow better provision of trauma care. However, data on acute and traumatic surgical conditions are scarce, therefore, making it difficult to influence governments on public health policies, and provision of both appropriate amenities and clinical resources. Economic evidence rather than speculations of economic impact from injuries needs to be presented to stakeholders before change can truly be made in low-resource settings.

Globalization comes at a cost. The coupling of cheaper vehicles and growing populations, with the lack of health care development and systems, fuel marginalization. Adequate planning of health systems in LMICs, in a low-cost manner, is essential for strengthening of services. A solid infrastructure will contribute to better patient care. There is an inherent lack of road traffic injury surveillance systems in LMICs. 16 Governments need to focus on establishing legislation and provision of adequate policing services which provide accountability when laws are broken.

Cost-effective trauma systems 4 that are contextualized to low-resource settings need to be further developed, as the “one size fits all” approach is not always constructive. Nevertheless, beyond the goals set by the WHO, 44 there should be a drive originating within nations through their health ministries to understand the short- and long-term benefits of providing safe and effective health care. Challenges will exist when changes to cultural norms are being implemented. Health economists should be consulted to see what funding is required for surgical conditions, and whether this puts strain on health budgets. Sustainability in health care reforms in combination with appropriate policies can make a robust health care system. Clinical governance strategies such as quality improvement and auditing are valuable tools which satisfy stakeholders and must be encouraged in LMIC health care systems. Additionally, corruption in global health agendas, and its impact on health and food security, is yet to be fully addressed.

Education throughout the training of health care professionals is paramount to obtain and maintain a level of good-quality trauma care. Traditionally, trauma and emergency surgery are not specifically taught in medical schools. However, this is gradually changing. For example, in parts of the United Kingdom, emergency surgery placements now exist for medical students. The concept of joint ventures between hospitals in LICs and HICs to improve education, training, and research would be advantageous. This is exemplified by the collaborative work between East, Central, and Southern African countries and Oxford University. 45 This partnership has enabled training in trauma management in nine countries in Africa with primary courses being delivered by the United Kingdom instructors and subsequently by local faculty. 45

Trauma registries are well developed and maintained in HICs. They play a significant role in the evaluation of patient care and assessing demographics for education. 46 47 Social media platforms, mobile apps, and radios are effective means of communication in LMICs for education and could be used to harness data by encouraging members of the public to report injuries. Nevertheless, these data collecting systems have to be monitored for to minimize incorrect and duplication of data. Technology in the form of mobile phones could also aid prehospital care and remote follow-up of patients.

## Conclusion

We acknowledge this review is limited by not being a systematic review of trauma in LMICs. However, it gives a synopsis of the key problems and provides possible solutions both novel and previously described in the literature. This review has delineated a range of economic and social strategies that are necessary for the provision of better trauma care in LMICs. Improvements in infrastructure along with education and training would likely lead to reduced morbidity and mortality among trauma patients in LMICs. This article also highlights a lack of research on trauma care in LMICs, more of which is needed for policy design and change.
